# TH-17 cells in rheumatoid arthritis

**DOI:** 10.1186/ar2477

**Published:** 2008-08-18

**Authors:** Shiva Shahrara, QiQuan Huang, Arthur M Mandelin, Richard M Pope

**Affiliations:** 1 Department of Medicine, Feinberg School of Medicine, Northwestern University 240 E. Huron, Suite M220, Chicago, IL 60611, USA; 2 Jesse Brown VA Chicago Healthcare System, 820 S. Damon, Chicago, IL 60612, USA

## Abstract

**Introduction:**

The aim of this study was to quantify the number of T-helper (TH)-17 cells present in rheumatoid arthritis (RA) synovial fluid (SF) and to determine the level of interleukin (IL)-17 cytokine in RA, osteoarthritis (OA) and normal synovial tissue, as well as to examine SF macrophages for the presence of IL-23, IL-27 and interferon (IFN)-γ.

**Methods:**

Peripheral blood (PB) mononuclear cells from normal and RA donors and mononuclear cells from RA SF were examined either without stimulation or after pretreatment with IL-23 followed by stimulation with phorbol myristate acetate (PMA) plus ionomycin (P/I). The abundance of TH-17 cells in RA SF was determined by flow cytometry. IL-17 levels were quantified in synovial tissue from RA, OA and normal individuals by ELISA and IL-23 was identified in SFs by ELISA. RA SF and control *in vitro *differentiated macrophages were either untreated or treated with the toll-like receptor (TLR) 2 ligand peptidoglycan, and then IL-23, IL-27 and IFN-γ mRNA levels were quantified by real-time polymerase chain reaction (RT-PCR).

**Results:**

Treatment with P/I alone or combined with IL-23 significantly increased the number of TH-17 cells in normal, RA PB and RA SF. With or without P/I plus IL-23, the percentage of TH-17 cells was higher in RA SF compared with normal and RA PB. IL-17 levels were comparable in OA and normal synovial tissues, and these values were significantly increased in RA synovial tissue. Although IL-17 was readily detected in RA SFs, IL-23 was rarely identified in RA SF. However, IL-23 mRNA was significantly increased in RA SF macrophages compared with control macrophages, with or without TLR2 ligation. IL-27 mRNA was also significantly higher in RA SF compared with control macrophages, but there was no difference in IL-27 levels between RA and control macrophages after TLR2 ligation. IFN-γ mRNA was also detectable in RA SF macrophages but not control macrophages and the increase of IFN-γ mRNA following TLR2 ligation was greater in RA SF macrophages compared with control macrophages.

**Conclusion:**

These observations support a role for TH-17 cells in RA. Our observations do not strongly support a role for IL-23 in the generation of TH-17 cells in the RA joint, however, they suggest strategies that enhance IL-27 or IFN-γ might modulate the presence of TH-17 cells in RA.

## Introduction

Interleukin (IL)-17 may play a critical role in the pathogenesis of rheumatoid arthritis (RA). IL-17 is capable of promoting inflammation by inducing a variety of pro-inflammatory mediators, including cytokines, chemokines and other mediators of bone and cartilage destruction in synovial fibroblasts, monocytes, macrophages and chondrocytes [1]. RA synovial explants spontaneously produce IL-17 [2] and increased levels of IL-17 are found in RA synovial fluid (SF) compared with osteoarthritis (OA) SF [3]. By immunohistochemistry, IL-17 has previously been identified in T lymphocytes in RA synovial tissue (ST), especially CD4+CD45RO+ T cells [2,3]. One subset of T cells, T helper (TH)1 cells, produce IFNγ and another subset, TH2 cells, produce IL-4 [4]; but as IFNγ and IL-4 are found at very low levels in the RA joint [5], the source of IL-17 in the RA joint was unclear until the recent discovery of a third subset of T cells capable of producing IL-17, the TH-17 cells. However, the abundance of TH-17 cells in the RA joint has not yet been fully characterised. Nonetheless, the potential importance of IL-17 in RA is supported by the observation that IL-17 is critical for the development of, and is an effective therapeutic target in, a variety of animal models of RA [6].

The mechanisms contributing to the development of TH-17 cells in humans have only been recently clarified. Several groups have demonstrated that IL-1β, IL-6, IL-23 and transforming growth factor (TGF)-β promote human TH-17 cell differentiation from naive peripheral blood (PB) CD4+ cells, resulting in the expression of IL-17 (also called IL-17A), IL-17F, IL-21, IL-22 and IL-6 [7,8]. Although it was initially suggested that the differentiation of human TH-17 cells is independent of TGF-β, recently published data demonstrate that the absence of TGF-β mediates a shift in T cell gene expression from a TH-17 profile to a TH1-like profile [7,8]. Others have shown that TGF-β and IL-21 uniquely promote the polarisation of TH-17 cells from human naive CD4+ T cells, and further that IL-1β together with IL-6 or IL-23 are only capable of inducing TH-17 cells from human memory CD4+ T cells [9].

Cell-cell contact of human CD4+ T cells with monocytes that have been activated by lipopolysaccarides (LPS) or peptidoglycan (PGN) promotes the development of TH-17 cells [10]. Consistent with a potential role in RA, IL-23 plays a major role in the pathogenesis of experimental arthritis, because IL-23-/- mice are resistant to the development of collagen-induced arthritis [11]. Conversely, IL-27 has recently been shown to suppress the development of TH-17 cells [12] and to suppress experimental arthritis [13]. Although IL-1 and IL-6 are highly expressed in the RA joint and IL-23 has been identified by immunohistochemistry in RA ST [14], the role of IL-23 is unclear, due to marked differences in the levels of IL-23 reported in previous studies [15-17]. Also, the expression of the cytokines IL-27 and IFN-γ, which might suppress TH-17 polarisation, have not been examined in RA SF.

In the present study, we document the presence of TH-17 cells in RA SF, and demonstrate that the abundance of TH-17 cells is significantly increased compared with RA or normal PB. Further, we show that RA ST express higher levels of IL-17 compared with OA and normal ST. We also demonstrate that IL-23 increases the abundance of TH-17 cells following short-term activation of mononuclear cells taken from RA SF, but not from normal PB or RA PB. Although IL-23 was rarely detected in RA SF, IL-23 mRNA was increased in RA SF macrophages compared with control macrophages, in the absence or presence of the toll-like receptor (TLR) 2 agonist, PGN. IL-27 was also increased in RA SF macrophages, although it was only modestly induced by PGN. While IFN-γ mRNA levels were undetectable in control macrophages, low levels were detected in RA SF macrophages and induction of IFN-γ mRNA by PGN was greater in RA SF macrophages compared with control macrophages. These observations support a role of TH-17 cells in the pathogenesis of RA, although the mechanisms responsible for the generation of these cells in the RA joint require further clarification.

## Materials and methods

### Patients

SFs were obtained from patients with RA, diagnosed according to the 1987 revised criteria of the American College of Rheumatology [18]. RA SF was obtained from 12 women and two men (mean age ± SE = 52.8 ± 6.3 years). Two patients were only taking prednisone (generic) (<10 mg/day) at the time of joint aspiration, seven patients were taking methotrexate (generic) plus an anti-tumor necrosis factor (TNF) 3 on an anti-TNF alone, one patient was only taking methotrexate, and one patient was taking azathioprine (generic) plus abatacept (Orencia, Bristol-Myers Squibb) Dosages vary widely by patient and were not tracked for the purposes of this study. Of the patients whose SFs were examined for cytokines (mean age = 52.6 ± 2.7 years), 15 were taking an anti-TNF, either alone (n = 1), with a non-biological disease-modifying anti-rheumatic drug (DMARD) (n = 10; methotrexate, leflunomide (generic) or azulfidine) or with low-dose prednisone (n = 4, <10 mg/day). Eight patients were taking low-dose prednisone, either alone (n = 4) or with methotrexate (n = 4). Four patients were taking no medication at the time of arthrocenthesis. RA PB was obtained from nine women (mean age 56.3 ± 7.5 years), of whom there were three taking methotrexate alone, two taking methotrexate plus an anti-TNF, one taking methotrexate and leflunomide, two taking methotrexate and abatacept and one patient taking rituximab (Rituxan, Genentech).

The studies were approved by the Northwestern University Institutional Ethics Review Board and all donors gave informed written consent.

### Cell isolation and culture

Macrophages were differentiated *in vitro *for seven days from monocytes, which were purified by elutriation from the PB mononuclear cells of healthy donors as previously described [19]. Heparinised SFs were centrifuged at 800 *g *at room temperature for 10 minutes to obtain cell-free SF. RA PB and RA SF mononuclear cells were isolated by Histopaque gradient centrifugation (Sigma-aldrich, St. Louis, MO, USA) RA SF macrophages were isolated by adherence for one hour, as previously described [19]. The control and RA SF macrophages were either untreated or treated with PGN (1 μg/ml) for four hours.

### Flow cytometric analysis of TH-17 cells

Mononuclear cells were either left untreated or incubated for 18 hours with IL-23 (20 ng/ml). Subsequently, the cells were incubated with PMA (50 ng/ml) and ionomyocin (1 μg/ml) plus brefeldin A (10 μg/ml) for four hours. Cells were blocked with 50% human serum and 0.5% bovine serum albumin in phosphate buffered saline and incubated with allophycocyanin-conjugated monoclonal anti-CD3 (eBioscience, San Diego, CA) for 30 minutes, fixed with 2% formaldehyde for 10 minutes, and then permeabilised with 0.1% NP40 for 10 minutes. Cells were then stained for fluorescein isothiocyanate labelled anti-CD4 and phycoerythrin-conjugated anti-IL-17 (eBioscience, San Diego, CA) or isotype control antibodies (eBioscience, San Diego, CA). TH-17 cells were identified as those that were CD3+CD4+IL-17+. The percentage of TH-17 cells in each sample was normalised for staining with its control IgG value by subtracting the percentage of cells that were positive when stained with the control IgG alone from the original percentage of TH-17 cells.

### Real-time PCR

Total cellular RNA was extracted using Guanidinium thiocyanate-phenol-chloroform extraction (Trizol reagent) (Invitrogen, Carlsbad, CA, USA), and reverse-transcription and RT-PCR were performed as previously described [19]. Relative gene expression was determined by the ΔΔC_t _method.

### Tissue homogenisation

Synovial tissues were homogenised as described previously [20] in 1 ml of complete Mini protease-inhibitor cocktail homogenisation buffer (Roche, Indianapolis, IN) on ice, followed by sonication for 30 seconds. Homogenates were centrifuged and filtered through a 0.45 μm pore size filter before quantifying the levels of IL-17 by ELISA. The final concentration of IL-17 in ST was normalised to the protein concentration in each tissue.

### Cytokine quantification

Human IL-23 (p19/p40) (eBioscience, San Diego, CA) and IL-17 (R&D Systems, Minneapolis, MN, USA) ELISA kits were used according to the manufacturers' instructions.

### Statistical analysis

The data were analysed using two-tailed Student's *t *tests for paired and unpaired samples. P values less than 0.05 were considered significant.

## Results

### TH-17 cells expressed in RA SF

**Figure 1:**
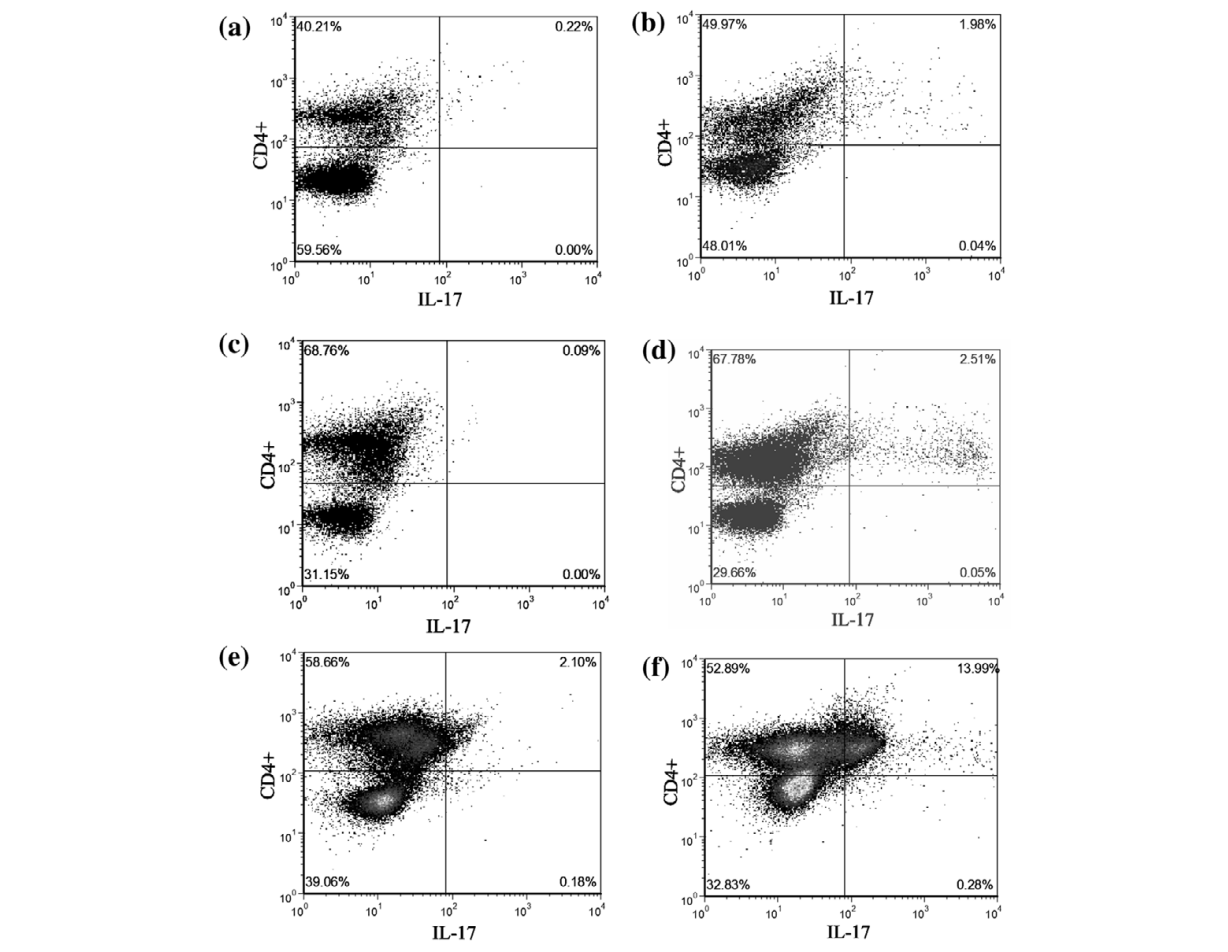
Identification of TH-17 cells in rheumatoid arthritis (RA) synovial fluid (SF), RA peripheral blood (PB) and normal PB. **(a, b) **Normal PB, **(c, d) **RA PB or **(e, f) **RA SF mononuclear cells treated with control medium (a, c, e) or IL-23 (20 ng/ml) for 18 hours, followed by the addition of PMA (50 ng/ml) plus ionomycin (1 μg/ml) for four hours (b, d, f), before immunostaining. The values are presented as mean ± SE of % CD4+IL-17+ cells within the total CD4 population.

**Figure 2:**
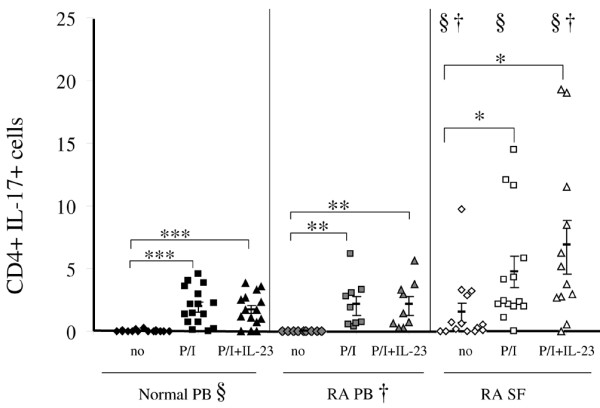
TH-17 cells are increased in rheumatoid arthritis (RA) synovial fluid (SF). Summary of flow cytometric analysis identifying the percentage of TH-17 cells in normal peripheral blood (PB) (n = 15–16), RA PB (n = 9) or RA SF cells (n = 12–14) that were untreated (no), treated with PMA plus ionomycin (P/I) only or P/I plus IL-23. The percentage of TH-17 cells in each sample was normalised to its IgG value. * represents p < 0.05, and *** represents p < 0.005 between the indicated groups. § or † at the top of the treatment groups for RA SF denotes significant differences (p < 0.05) compared with normal or RA PB, treated in the same way.

In the absence of stimulation, the percentage of TH-17 cells (CD3+CD4+IL-17+) was higher (p < 0.05) in RA SF (1.5 ± 0.72%) compared with RA PB (0%) and normal PB (0.04 ± 0.02%) (Figures 1 and 2). Short-term activation with PMA plus ionomycin (P/I) was used to enhance detection of IL-17 within the CD4+ T cells, and not to promote polarisation. Treatment with P/I alone significantly increased the number of TH-17 cells in normal PB (0.04% to 1.9 ± 0.4%, p < 0.005), RA PB (0% to 2.1 ± 0.6%, p < 0.05) and RA SF (1.5% to 4.7 ± 1.24%, p < 0.05) (Figure 2). Mononuclear cells were also incubated with control medium or with IL-23 for 18 hours before P/I was added. Pretreatment of RA SF mononuclear cells with IL-23 and P/I resulted in a significantly (p < 0.05) increased frequency of TH-17 cells (6.8 ± 1.93%) (Figures 1f and 2), compared with untreated RA SF (Figures 1e and 2). Employing normal PB (1.7 ± 0.3%) or RA PB (2.1 ± 0.6%), pretreatment with IL-23 did not increase the number of TH-17 cells compared with P/I alone; however, the percentage of TH-17 cells was significantly higher than in the untreated group. Following treatment with IL-23 plus P/I, the abundance of TH-17 cells in RA SF was significantly (p < 0.05) greater than observed with normal PB and RA PB treated in the same fashion (Figures 1b, 1f and 2). In conclusion, these results demonstrate the increased abundance of TH-17 cells in RA SF compared with normal PB and RA PB.

### Level of IL-17 in RA, OA and normal synovial tissues

**Figure 3:**
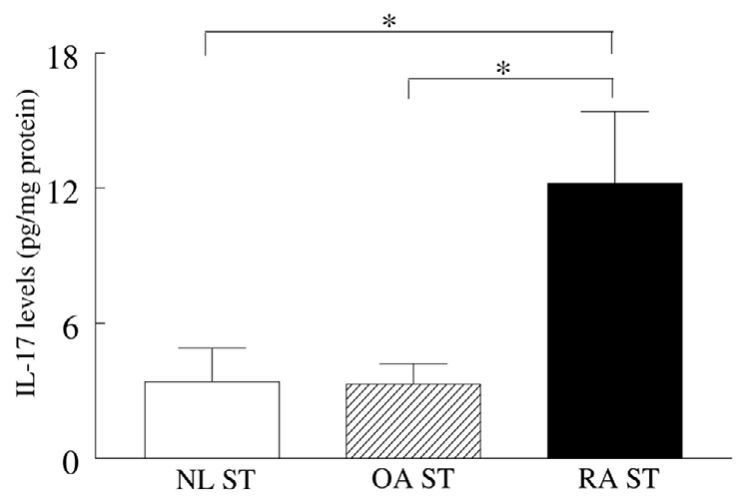
IL-17 expression is higher in rheumatoid arthritis (RA) synovial tissue (ST) compared with OA and normal ST. STs from RA (n = 10), osteoarthritis (OA) (n = 9) and normal (NL) (n = 6) individuals were homogenised, centrifuged and filtered before quantifying the levels of IL-17 by ELISA. The final concentration of IL-17 in STs was normalised to the protein concentration in each tissue. The results are shown as mean ± SE. *p < 0.05.

Since OA SFs do not contain enough cells to quantify TH-17 cell number, we therefore measured the levels of IL-17 in RA ST, OA ST and normal ST. Our results demonstrate that OA ST (3.3 ± 0.9 pg/mg) and normal ST (3.4 ± 1.5 pg/mg) have comparable levels of IL-17, and that the levels of IL-17 were 3.5-fold increased (p < 0.05) in the RA ST (12.2 ± 3.2 pg/mg) (Figure 3).

### IL-23, IL-27 and IFN-γ in the RA joint

**Figure 4:**
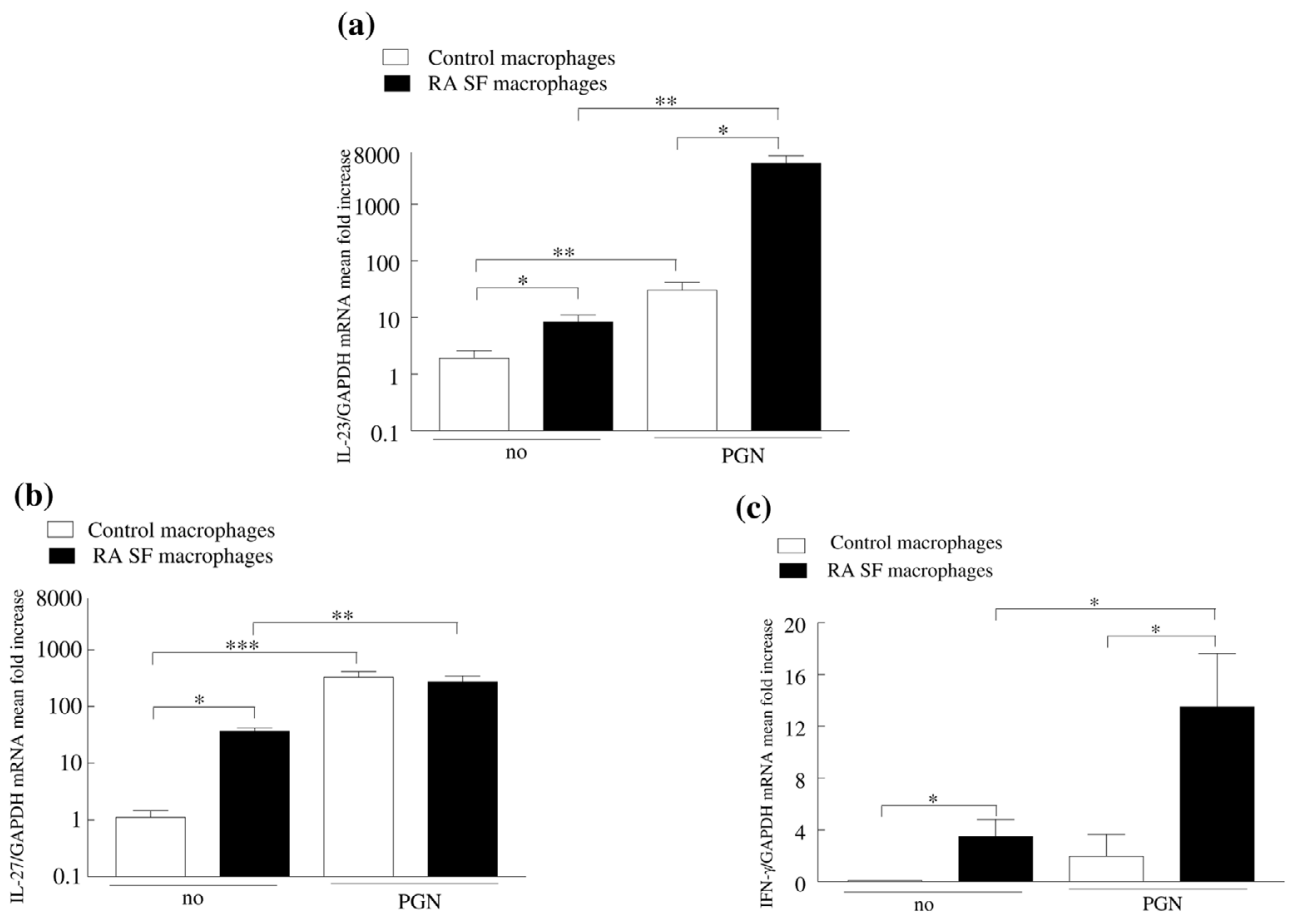
Rheumatoid arthritis (RA) synovial fluid (SF) macrophages express increased IL-23, IL-27 and IFN-γ. mRNA was extracted from RA SF macrophages (n = 12) and control macrophages (n = 12) that were either untreated or treated with peptidoglycan for four hours. Real-time PCR was employed to identify mRNA for **(a)** IL-23, **(b)** IL-27 and **(c)** IFN-γ which were normalised to glyceraldehyde 3-phosphate dehydrogenase (GAPDH). The results are presented as fold increase compared with control macrophages for IL-23, IL-27 and RA SF macrophages for IFN-γ, and represent the mean ± SE. *p < 0.05, **p < 0.01, ***p < 0.005.

RA SF T lymphocytes were responsive to IL-23, so the SFs from 28 patients with RA were examined for the presence of IL-23, employing an assay specific for IL-23p19/p40. However, IL-23 was only detected in four samples (20, 24, 30 and 66 pg/ml), while none of the SFs from patients with OA were positive (data not shown). In contrast, IL-17 was detected in all the RA SFs (233 ± 64 pg/ml) at levels significantly greater than those observed in OA SF (38 ± 18 pg/ml). Despite the paucity of IL-23 in RA SF, macrophages from the joints of patients with RA expressed 4.3-fold (p < 0.05) more IL-23 mRNA compared with control *in vitro *differentiated macrophages (Figure 4a). Following activation with PGN, there was a 15-fold increase of IL-23 mRNA in the control macrophages, but a significantly greater increase (p < 0.01) of 621-fold in the RA SF macrophages.

Since IL-27 and IFN-γ may suppress the development of TH-17 cells, macrophages were also examined for IL-27 and IFN-γ mRNA. Like IL-23, IL-27 and IFN-γ mRNA levels were significantly higher in RA SF macrophages compared with control macrophages (Figures 4b, c). Following treatment with PGN, the IL-27 and IFN-γ mRNA increased in both RA SF (7.5-fold for IL-27 and 3.8-fold for IFN-γ) and control macrophages (295-fold for IL-27 and from undetectable to 1.9-fold for IFN-γ). Similar to IL-23, the levels of IFN-γ were significantly higher after PGN stimulation of macrophages from RA SF compared with control macrophages. In contrast to the results observed with IL-23, after activation with PGN, there was no difference in IL-27 mRNA between the patients and the controls. These studies demonstrate that although IL-23 is not plentiful in RA SF, it is expressed in RA synovial macrophages and is greatly increased after TLR2 ligation. While IL-27 and IFN-γ are expressed in RA SF macrophages, their induction after TLR2 ligation was less compared with the induction of IL-23 in RA SF macrophages.

## Discussion

In the present study, we demonstrate that TH-17 cells are more abundant in RA SF compared with normal PB and RA PB. IL-17 is produced by activated human CD4+CD45RO+ memory T cells, while only low levels of IL-17 are secreted by CD4+CD45RA+ naive T cells [21,22]. The increased presence of TH-17 cells in RA SF may be related to an increased percentage of memory CD4+ T cells in RA SF. However, this is not likely to be the entire explanation because about 50% of normal PB and RA PB CD4+ cells are memory cells [23]. In the absence of IL-23 and P/I, TH-17 cells were 38-fold higher in RA SF compared with normal PB, whereas TH-17 cells were essentially undetectable in RA PB. The reduction of TH-17 cells in RA compared with normal PB may be due to the enrichment of TH-17 cells in the RA joint, although differences in disease activity or treatment may also be responsible. Our observations contrast with a recently published study that observed a reduction of TH-17 cells in RA SF compared with RA PB [24]. The reason for the difference between that study and other studies concerning IL-17 and TH-17 cells (including the work presented here) is not clear, although it is possible that differences in patient selection and therapy may have contributed, as well as technical differences such as the antibodies used or methods employed for cell fixation or permeabilisation.

Treatment with IL-23 plus P/I resulted in increased numbers of TH-17 cells in RA SF, which were greater than those observed in normal and RA PB. The expression of IL-23R is increased on CD45RO memory T cells, and IL-23 may also induce the expression of its own receptor [21,22]. Although our data suggest that RA SF CD4+ cells are capable of responding to IL-23, the role of this cytokine in TH-17 cell polarisation in RA remains uncertain. We detected IL-23 in only four of the 27 RA SFs analysed, while all the RA SFs possessed IL-17. In marked contrast, a recent study showed very high levels of IL-23p19 in RA SF and PB [16]. The explanation for the very high values [16] may be technical; however, patient characteristics and therapy at the time of obtaining the SFs may be important. In this regard, a *post hoc *examination of the clinical features of the four RA patients in the present study with high SF levels of IL-23 revealed that all four were on anti-TNF treatment, suggesting at least a moderate level of RA burden in order to clinically merit these expensive agents. Further, three of the four had documented lengthy disease duration of between 12 and 25 years (the fourth also had 'longstanding' disease according to the treating physician's narrative notes), and three of the four had a well-documented history of repeated presentation to their treating physician for recurrent and/or unremitting swelling in the knees (which are the joints from which the SF samples in this study were taken) despite being on anti-TNF treatment.

Nonetheless, our observations are consistent with a recent study that also observed low levels of IL-23 in RA SF [25]. Another study demonstrated detectable levels of IL-23 in about half of RA SFs examined, with only two samples possessing more than 250 pg/ml, which was more consistent with our observations [15]. Suggesting the importance of therapy in the expression of IL-23, these authors demonstrated a significant correlation between the levels of IL-23 and IL-17 in the RA SF before the initiation of etanercept [15]. Consistent with these observations, the two patients in the current study who had the greatest increase of TH-17 cells after treatment with P/I plus IL-23 were taking no DMARDs or biological agents. Further, in the RA joint, CD4+ cells may be more responsive to low levels of IL-23, especially in an environment rich in IL-1β and IL-6, as observed in the RA joint.

Even though IL-23 p19/p40 was very low in RA SF, macrophages isolated from RA SF had significantly increased IL-23 p19 mRNA expression (four-fold increase) compared with control macrophages. Consistent with this observation, employing immunohistochemistry, IL-23p19 was expressed abundantly in RA ST [14,25]. Further, RA SF macrophages stimulated with PGN expressed significantly higher levels of IL-23 mRNA compared with control macrophages treated similarly. The higher levels of IL-23 mRNA in RA SF versus control macrophages may be due to increased expression of TLR2 on macrophages obtained from the RA joint and the expression of endogenous TLR ligands in the RA joint [19]. Supporting the relevance of this possibility, TLR ligand-activated monocytes and dendritic cells result in TH-17 polarisation [10,26].

The levels of IL-27 mRNA were also significantly higher in RA SF compared with control macrophages; however, the levels of IL-27 were similar in both groups in the presence of the TLR2 ligation. Since IL-27 may be important in suppressing the differentiation of TH-17 cells [12], these observations suggest that therapy directed at enhancing the expression of IL-27 in RA may be therapeutically beneficial. Supporting this approach, others have shown that IL-27 ameliorates collagen-induced arthritis [13], although IL-27 promoted proteoglycan-induced arthritis [27].

IFN-γ in RA SF macrophages was also examined because IFN-γ may suppress TH-17 polarisation. IFN-γ mRNA was slightly increased in RA SF, compared with control macrophages. The induction of IFN-γ was significantly greater after TLR2 ligation employing RA SF, compared with control macrophages. The expression of IFN-γ in human alveolar macrophages has been reported in sarcoidosis and after infection with *Mycobacterium tuberculosis *[28,29]. These results indicate that RA SF contains multiple factors that may modulate the development of TH-17 cells in the RA joint.

## Conclusion

In summary, these observations support the role of TH-17 cells in the pathogenesis of RA. However, the role for IL-23 in established disease is less clear. Further, therapy aimed at enhancing IL-27 or IFN-γ may be an attractive approach to suppress TH-17 cell polarisation in RA.

## Abbreviations

DMARDs = disease-modifying anti-rheumatic drugs; IFN = interferon; IL = interleukin; OA = osteoarthritis; PB = peripheral blood; PGN = peptidoglycan; P/I = PMA (phorbol myristate acetate) plus ionomyocin; RA = rheumatoid arthritis; RT-PCR = real-time polymerase chain reaction; SF = synovial fluid; ST = synovial tissue; TGF = transforming growth factor; TH = T helper; TLR = toll-like receptor; TNF = tumor necrosis factor.

## Competing interests

The authors declare that they have no competing interests.

## Authors' contributions

SS was responsible for the design of the study, acquisition of data, analysis and interpretation of the data, and manuscript preparation. QQH and AMM were responsible for acquisition of data and manuscript preparation. RMP was responsible for the design of the study, interpretation of the data and manuscript preparation. All authors have approved the content of the manuscript.
